# Molecular Weights of Bovine and Porcine Heparin Samples: Comparison of Chromatographic Methods and Results of a Collaborative Survey

**DOI:** 10.3390/molecules22071214

**Published:** 2017-07-19

**Authors:** Sabrina Bertini, Giulia Risi, Marco Guerrini, Kevin Carrick, Anita Y. Szajek, Barbara Mulloy

**Affiliations:** 1 Istituto di Ricerche Chimiche e Biochimiche “G. Ronzoni” via G. Colombo 81, 20133 Milano, Italy; bertini@ronzoni.it (S.B.); giulia.risi02@universitadipavia.it (G.R.); guerrini@ronzoni.it (M.G.); 2 Department of Chemistry, University of Pavia, viale Taramelli 12, 27100 Pavia, Italy; 3 U.S. Pharmacopeial Convention (USP), 12601 Twinbrook Parkway, Rockville, MD 20852, USA; klc@usp.org (K.C.); anita.szajek@nih.gov (A.Y.S.); 4 Center for Scientific Review (CSR), National Institutes of Health, 6701 Rockledge Dr. Rm. 4187, Bethesda, MD 20892, USA; 5 Institute of Pharmaceutical Science, Franklin Wilkins Building, King’s College London, 150 Stamford St., London SE1 9NH, UK

**Keywords:** bovine heparin, porcine heparin, molecular weight, size exclusion chromatography, pharmacopeia

## Abstract

In a collaborative study involving six laboratories in the USA, Europe, and India the molecular weight distributions of a panel of heparin sodium samples were determined, in order to compare heparin sodium of bovine intestinal origin with that of bovine lung and porcine intestinal origin. Porcine samples met the current criteria as laid out in the USP Heparin Sodium monograph. Bovine lung heparin samples had consistently lower average molecular weights. Bovine intestinal heparin was variable in molecular weight; some samples fell below the USP limits, some fell within these limits and others fell above the upper limits. These data will inform the establishment of pharmacopeial acceptance criteria for heparin sodium derived from bovine intestinal mucosa. The method for MW determination as described in the USP monograph uses a single, broad standard calibrant to characterize the chromatographic profile of heparin sodium on high-resolution silica-based GPC columns. These columns may be short-lived in some laboratories. Using the panel of samples described above, methods based on the use of robust polymer-based columns have been developed. In addition to the use of the USP’s broad standard calibrant for heparin sodium with these columns, a set of conditions have been devised that allow light-scattering detected molecular weight characterization of heparin sodium, giving results that agree well with the monograph method. These findings may facilitate the validation of variant chromatographic methods with some practical advantages over the USP monograph method.

## 1. Introduction

Heparin preparations for medical use are polydisperse polymers derived from mast cell containing tissues, with molecular weights determined by both the tissue of origin and the processes involved in heparin manufacture [1]. Measurement of the molecular weight distribution of heparin samples usually involves size exclusion chromatography (SEC) with some form of light scattering detection, as this technique does not depend on calibrant reference materials [2,3]. A chromatographic method was developed for inclusion as an identification method in the USP monograph for heparin sodium [4], using a sample of porcine mucosal heparin as a broad standard calibrant, characterized in a collaborative study involving eight laboratories. Though all these laboratories were expert in light scattering detection, reproducibility of results between laboratories was not strong. However, it was possible to combine the experimental results of this study to give a characteristic molecular weight distribution for the calibrant material, expressed as a slice table allowing the use of the calibrant as a broad standard (the USP Heparin Sodium Molecular Weight Calibrant (Reference Standard) RS) [5].

Only heparin from porcine intestinal mucosa (porcine mucosal heparin) is currently approved for use in the USA, but the US Food and Drugs Administration (FDA) is investigating the possibility of introduction of heparin sodium from bovine intestinal mucosa (bovine mucosal heparin) into the US market [6]. Bovine mucosal heparin is licensed for use in several countries in the world. As porcine and bovine heparin differ in many aspects of their structure and activity [7,8,9,10], data have been collected on the potency and physicochemical characteristics of bovine heparin lots in current use in these countries. The USP will use the collected data to generate proposed acceptance criteria for potency and identification assays including molecular weight average and distributional parameters.

A multi-laboratory collaborative study has therefore been organized by the USP to obtain consensus molecular weight data using the USP Heparin Sodium monograph method [4,5] for a panel of bovine heparin samples in comparison with standard samples of porcine mucosal heparin. In addition to its data-gathering role for specification setting, this set of samples provides an opportunity to investigate further the discrepancies between light scattering methods and the USP calibrant-based method, and to determine whether alternative column types, as compared with those referred to in the USP Heparin Sodium monograph, might have acceptable chromatographic properties for heparin sodium MW determination either using light scattering detection or with the use of the USP Heparin Sodium Molecular Weight Calibrant RS.

### Summary and Aims of the Study

Phase 1: Molecular weight parameters as described in the USP Heparin Sodium monograph [4] were measured in six laboratories using the method described in the USP Heparin Sodium monograph [4] for 20 lots of bovine mucosal heparin, 2 lots of bovine lung heparin, and 2 standard samples of porcine mucosal heparin. The data collected in this phase of the study will contribute to the establishment of suitable acceptance criteria for the molecular weight distribution of bovine mucosal heparin sodium.

Phase 2: Using the same heparin samples as Phase 1, a single laboratory assessed results from 12 distinct chromatographic methods, varying in column type, mobile phase, and calibration method. The aim of this phase of the study was to identify alternative chromatographic methods giving similar molecular weight values to those obtained using the USP Heparin Sodium monograph method, avoiding some practical disadvantages of the USP method.

## 2. Results and Discussion

### 2.1. Phase 1: Collaborative Survey of Bovine and Porcine Heparin Samples

Results for 10 heparin samples were obtained from six participating laboratories, and for a further 14 samples from five laboratories. The sample codes are listed in Table S1 (Supplementary Materials), with species and tissue of origin; there were 20 samples of heparin manufactured from bovine intestinal mucosa (bovine mucosal heparin), two samples of bovine lung heparin, and two samples of heparin manufactured from porcine intestinal mucosa (porcine mucosal heparin). System suitability requirements described in Materials and Methods (below) were met for each laboratory on each day of the study.

An earlier study of heparin samples from porcine intestinal mucosa, currently the only acceptable source for heparin sodium in the USA, was used to derive appropriate acceptance criteria for molecular weight distribution by setting limits to the weight-average molecular weight *M_w_* (15,000 to 19,000 g/mol), *M_24,000_* (no more than 20%) and the ratio *M_8000–16,000_*/*M_16,000–24,000_* (no less than 1.0) [5].

**Figure 1:**
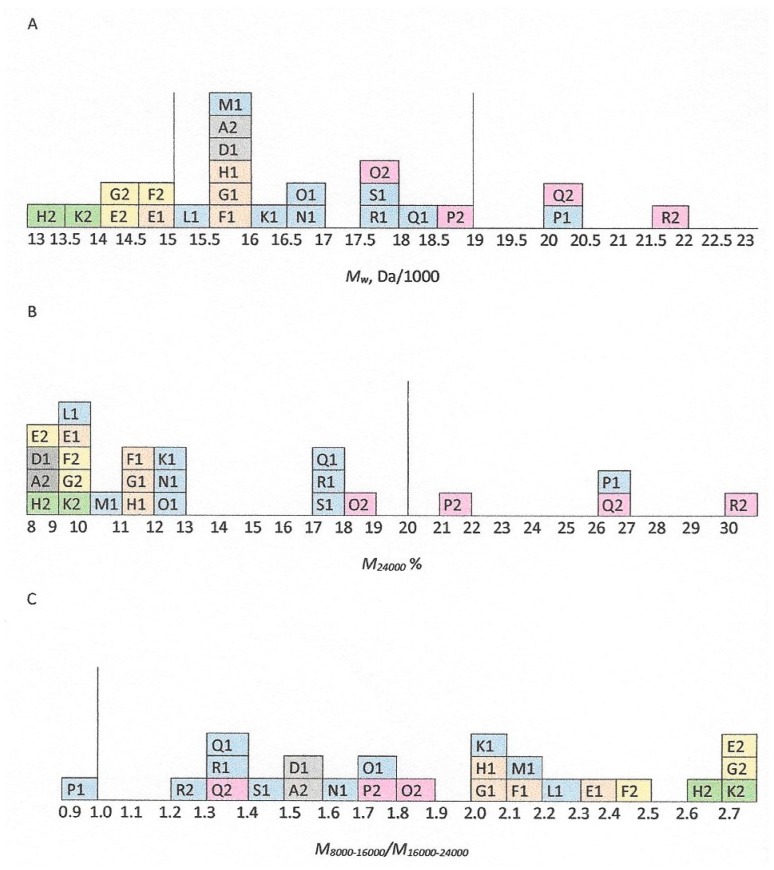
Histogram plots of Phase 1 summary results for 24 heparin samples (see Table S1) (**A**) *M_w_*, (**B**) *M_24,000_* and (**C**) *M_8000–16,000_*/*M_16,000–24,000_* for bovine lung heparin (green), porcine mucosal heparin (grey), and bovine mucosal heparin from sample donors 1 (orange), 3 (blue), 4 (pink), and 6 (yellow). Values recorded are the mean values from five or six laboratories (see Table S2A–C) The vertical lines indicate upper and lower limit acceptance criteria in the USP monograph for heparin sodium.

These molecular weight characteristics for all 24 heparin samples, as determined in the participating laboratories, are listed in Table S2A (*M_w_*), Table S2B (*M_24,000_*) and Table S2C (*M_8000–16,000_/M_16,000–24,000_*) (Supplementary Materials) and summarized in Figure 1.

The two porcine mucosal heparins A-3 and D-1 have *M_w_* near 16,000 Da, a typical value for heparin sodium as previously determined [5] and close to the characteristic value of 16,000 Da for the USP Heparin Sodium Identification RS. They have *M_24,000_* of about 9%, and the ratio *M_8000–16,000_*/*M_16,000–24,000_* is about 1.5. Two bovine lung heparins H-2 and K-2 have lower *M_w_*, outside the acceptable range for heparin sodium at about 13,500 Da. This is consistent with values determined for bovine lung heparins dating from the 1950s to the 1990s [11]. 

For the 20 bovine mucosal heparins, variability between individual samples is similar to that for porcine heparin before introduction of molecular weight acceptance criteria, as determined previously [5]. Three samples out of 23 (P-1, Q-2, and R-2) have *M_w_* higher than the top limit of 19,000 Da, and the same three samples have more than the limit of *M_24,000_* (20%). One of these samples also has a low value for the ratio *M_8000–16,000_*/*M_16,000–24,000_*, though the mean value from five laboratories rounds up to 1.0, the lower limit for this value. Figure 1 is shaded to indicate the donor numbers of each sample, and systematic differences can be seen in the molecular weight profiles of samples from each source. These differences are likely to originate from variant manufacturing protocols, though variations in the source tissues due to climate, nutrition, etc. are also possible.

**Figure 2:**
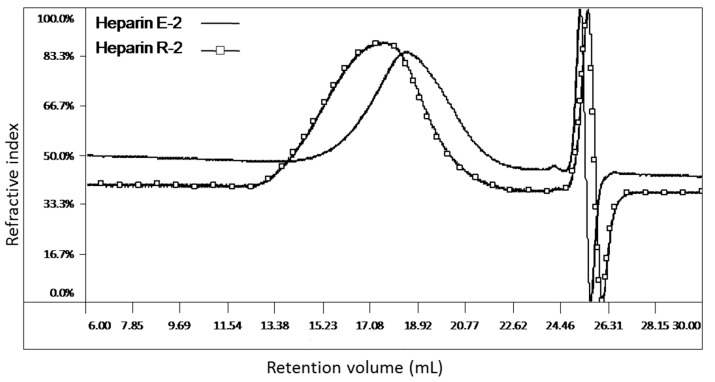
Phase 1: Molecular weight distributions for the samples of bovine mucosal heparin with highest (R-2) and lowest (E-2) molecular weights as measured by the USP Heparin Sodium monograph method. Chromatographic profiles refer to Method 1.

Figure 2 illustrates RI-detected chromatograms of the two bovine heparin samples with the lowest (E-2) and highest (R-2) values of *M_w_*. Though there is considerable overlap between the two samples, it is clear that sample E-2 contains a major amount of low molecular weight material, with longer retention time, compared with sample R-2. At higher molecular weights (short retention time), sample R-2 has about 30% material over 24,000 Da (well outside the USP’s acceptable range for porcine heparin sodium) whereas sample E-2 only has about 8% (values of *M_24,000_* from Table S2B). Values for *M_8000–16,000_*/*M_16,000–24,000_* reflect the molecular weight distribution in the mid-range; this ratio varies from 2.70 for sample E-2 to 1.28 for sample R-2 (Table S2C). 

### 2.2. Phase 2: Comparison of Different Chromatographic Methods

20 Samples of bovine mucosal heparin, two samples of bovine lung heparin, two samples of porcine mucosal heparin and one USP Heparin Sodium Identification RS were analyzed in different chromatographic conditions (see Table S5) by one laboratory involved in the project, with the purpose of comparing the conventional USP calibration method to a light-scattering method; furthermore, an evaluation of silica (two column sets, called A and B) and polymeric columns (two column sets, called C and D) chromatographic performances was done to determine whether polymeric columns might have acceptable chromatographic properties for heparin sodium MW determination (method details are reported below and in the Supplementary Material). Actually, most of the pharmacopeia chromatographic assays for the molecular weight distributions of heparin use silica columns, for which the great advantage of high resolution is countered by very short life time and many problems of compatibility with samples (interactions, pH, and so on).

**Figure 3:**
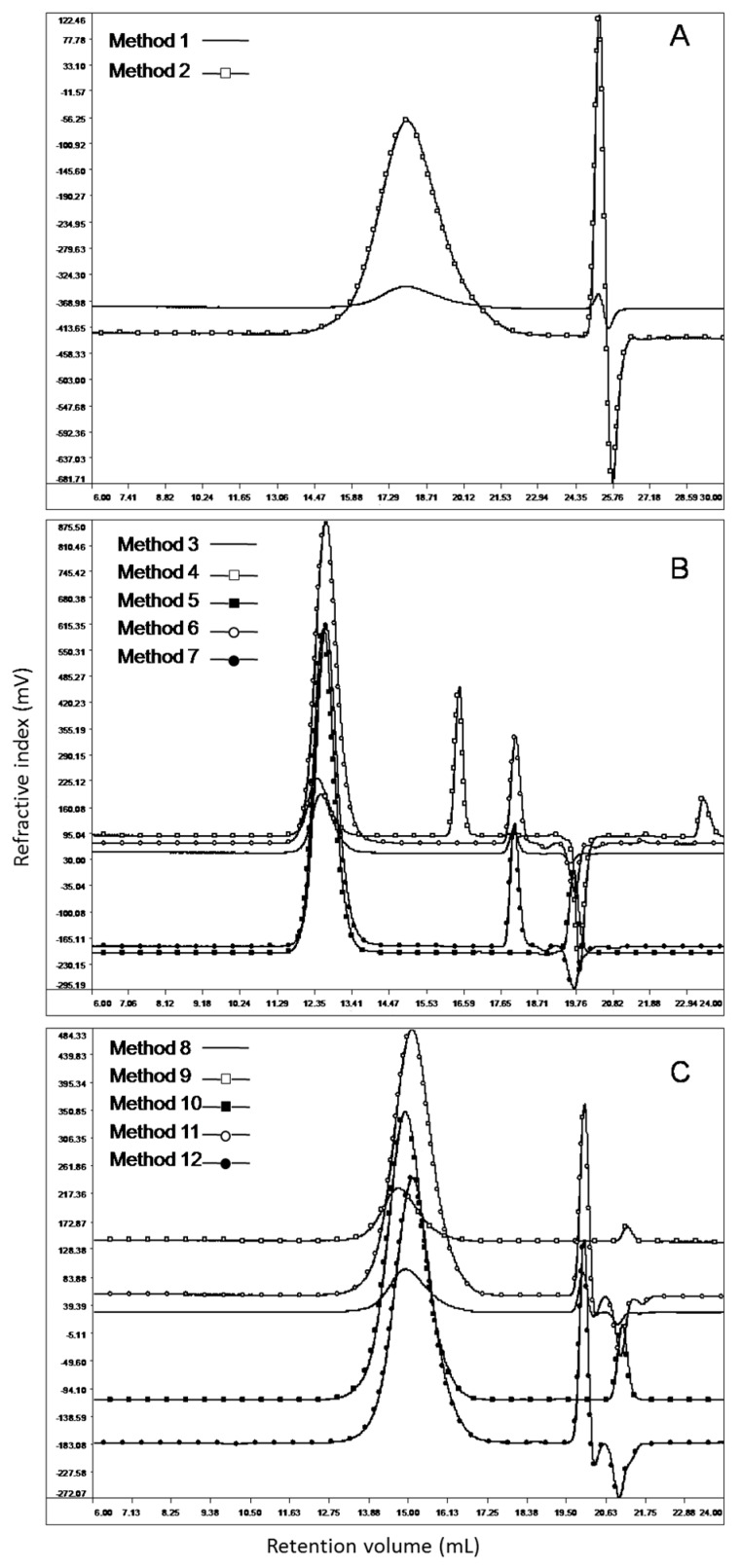
Phase 2: Overlay view of USP Heparin Sodium Identification RS chromatographic profiles in 12 distinct chromatographic systems. Panels: (**A**) Methods 1 and 2; (**B**) Methods 3–7; (**C**) Methods 8–12.

After first analysis with silica columns sets A and B (respectively Methods 1 and 2), samples were analyzed with polymer column set C (Methods from 3 to 7) and finally with polymer column set D (Methods from 8 to 12). The chromatographic profiles overlay of the USP Heparin Sodium Identification RS is reported in Figure 3. As can be observed, the elution peak is well separated from the mobile phase peak, so all chromatographic conditions tested are suitable for the analysis of this sample. Same results were obtained for the analysis of the other samples (chromatograms not reported). After acquisition, data were elaborated using suitable GPC software, as described in Supplementary Material section. Results for weight-average molecular weight *M_w_* of the heparin samples, percent proportion of material *M_24,000_* and the ratio *M_8000–16,000_*/*M_16,000–24,000_* are reported in Table S3A–C of the Supplementary Material section; results obtained for the analysis of the USP Heparin Sodium Identification RS are reported in Table 1.

The weight-average molecular weight *M_w_* range calculated for the USP Heparin Sodium Identification RS, as measured to assess system suitability (see Methods section), is between 15,200 and 17,700 Da, with an average value of 16,200 Da. Acceptance criteria for the system suitability test indicate that *M_w_* for this sample should lie within +/−500 Da of the established value of 16,000 Da (see Methods for the full set of system suitability requirements). Results for Methods 2, 4, 5 and 7, respectively 17,700, 15,200, 16,700 and 17,000 Da are out of the USP acceptance criteria, but considering all 12 methods *M_w_* results, the RSD% calculated is 4.17%, an acceptable value taking into account the instrument sensitivity and the different chromatographic conditions tested. One of the most critical points in the analysis with light-scattering detector is the use of the correct dn/dc value, a parameter that is a function not only of the sample but also of the chromatographic conditions, and this is the base of the differences observed in molecular weight distribution results [5]. The values used in this work were experimentally calculated for a heparin sodium sample by the laboratory involved in the comparison of chromatographic methods.

More in detail: Methods 5 and 7 were acquired at 40 °C with same columns and both gave high molecular weight and of course higher *M_24,000_* in comparison with Method 1 (official USP method for the analysis of heparin sodium molecular weight distribution); instead Methods 3, 4 and 6, acquired at 30 °C with the same columns but different calibration, gave lower molecular weight in comparison to 5 and 7 (although Methods 3 and 6 respects USP acceptance criteria). So, it is clear that column set C does not completely have the acceptable chromatographic properties required. The main reason of these differences could be the different particle size between columns sets C and A (both columns of the set C have a particle size of 7 μm, while columns set A have a particle size of 5 and 8 µm). Regarding to the columns set B, both molecular weight and ratio *M_8000–16,000_*/*M_16,000–24,000_* are out of the acceptance criteria; again, the main problem could be the particle size of the columns set (5 µm). As a conclusion for this first part, it seems that with a chromatographic system in which the particle size is the same for each column used in series, the correct resolution required is not reached, with the only exception of Method 3 chromatographic conditions, very similar to the USP official method. Looking at Methods 8 to 12, each of these chromatographic conditions results are within the acceptance criteria; the fact that column set D particle sizes are different (respectively 10 µm for the TSKG4000SWXL and 7 μm TSKG3000PWXL) could be a confirmation that having a single particle size in a column set cannot reach the right resolution required. Particularly remarkable is that the use of a light-scattering detector allows us to reach results comparable with the official USP method for the analysis of heparin sodium.

**Figure 4:**
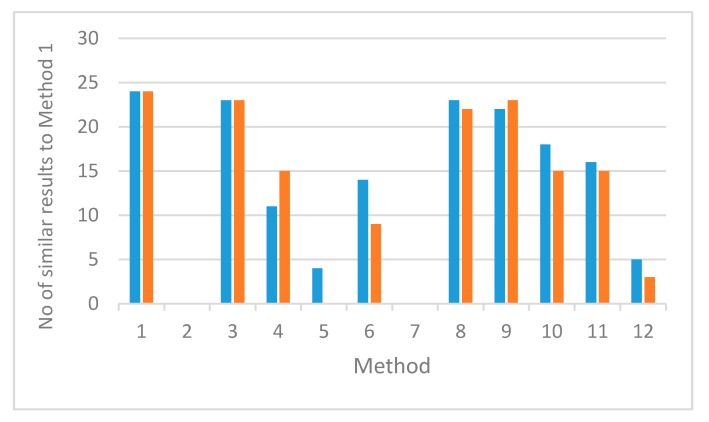
Phase 2: Column chart indicating similarity in molecular weight results for 24 heparin samples, between the USP Heparin Sodium monograph method (Method 1) and 11 other distinct chromatographic methods (Table S5). Blue columns plot *M_w_* data and orange columns plot *M_24,000_*. Data are taken from Table S3A,B; similarity criteria are for *M_w_*, values differ from Method 1 by less than 500 Da; for *M_24,000_* values differ from Method 1 by less than 10%.

Taking into account results from the whole set of 24 heparin samples, values for *M_w_* (Table S3A) and *M_24,000_* (Table S3B) can be used to compare methods for use over a wide range of heparin samples (Figure 4). Methods 3, 8 and 9 give results in especially good agreement with Method 1 over the entire range; few values for *M_w_* are more than 500 Da away from the Method 1 value, and few values for *M_24,000_* are outside +/−10% of the Method 1 value.

Methods 10 and 11 use the same column set as Methods 8 and 9, but use the Broad Standard calibrant (The USP Heparin Sodium Molecular Weight Calibrant RS). This calibrant was characterized for use with the L59 silica columns specified in the monograph. The calibrant information for a broad standard is a slice table of mass fraction vs. molecular weight (for example Table S4), which is a property of the calibrant material, theoretically independent of the chromatographic system used. In principle therefore, the broad standard calibrant should be transferable to any SEC column for which the sample is fully included. Methods 10 and 11 give reasonable agreement with Method 1 across most of the range of heparin samples in the panel, but less good agreement than do light-scattering Methods 8 and 9. Transference of this calibration method to column types other than those specified in the monograph cannot be guaranteed, and is particularly poor for column set C in this study (see Figure 4, Methods 5, 6 and 7), but appears to work best when used for heparin samples with molecular weight distributions within the current USP acceptance criteria (Table S3A,B).

## 3. Materials and Methods

### 3.1. Materials

USP Heparin Sodium Molecular Weight Calibrant and USP Heparin Sodium Identification RS were provided by USP. Twenty heparin sodium samples from bovine intestinal mucosa, and two from bovine lung were donated by four manufacturers of heparin; two standard samples from porcine intestinal mucosa were from USP and NIBSC. Sample codes are listed in Table S1.

### 3.2. Phase 1: The Collaborative Study

For the collaborative study, participants followed protocols based on the USP Heparin Sodium Monograph Identification Test D: Molecular weight determinations [4]. Briefly, the samples were analyzed by SEC on silica-based size exclusion columns USP code L59 (for example, a TSK G4000 SWXL (7.8 mm × 30 cm) and a TSK G3000 SWXL column (7.8 mm × 30 cm) in series, preceded by a TSK SWXL guard column; Tosoh Bioscience) using 0.1 M ammonium acetate (with 0.02% sodium azide preservative) as a mobile phase at a flow rate of 0.6 mL/min. Detection was by refractive index (RI) increment; the columns and detector were maintained at 30 °C. The calibrant, system suitability sample, and all heparin samples were taken up at 5 mg/mL in mobile phase; injection volume was 20 µL. Duplicate determinations were performed for each heparin sample in each laboratory, and results were reported to the USP.

#### Analysis of the Chromatographic Data

Broad standard calibration was performed using a chromatogram of the USP Heparin Sodium Molecular Weight Calibrant RS, baseline corrected and integrated; the cumulative area at each point under the heparin peak was calculated. Using the broad standard table (Table S4), points in the chromatogram were identified for which the percent cumulative area was closest to the percent fractions listed in the table; the molecular weight (MW) in the table was then assigned to the corresponding retention time (RT) in the chromatogram. For the set of retention times and molecular weights identified, log(MW) vs. RT was fitted to a third-order polynomial function. For each injection of the heparin samples and the system suitability sample, the weight average molecular weight *M_w_* was calculated according to the formula

(1) $${\bar{M}}_{w}=\frac{\sum_{i}RI_{i}M_{i}}{\sum_{i}RI_{i}}$$

where *RI_i_* is detector response at each point *i* and *M_i_* is molecular weight at each point *i*.

Proportions of material within specific molecular weight ranges were calculated as follows: the percentage of heparin with molecular weight in the range 8000 to 16,000 Da, *M_8000–16,000_*, the percentage of heparin with molecular weight in the range 16,000 to 24,000 Da, *M_16,000–24,000_*, and the percentage of heparin with molecular weight greater than 24,000 Da, *M_24,000_*.

System suitability criteria were as follows, taken from the USP Heparin Sodium monograph: in the chromatogram of the system suitability sample (the USP Heparin Sodium Identification RS), there is a baseline resolution between the heparin and salt peaks. The linear regression coefficient of the calibration curve fitted to the broad standard table values must be not less than 0.990 in magnitude, using a third order polynomial equation. The mean of the calculated *M_w_* from the duplicate injections of system suitability solution rounded up to the nearest 100 Da is within 500 Da of the assigned value of 16,000. The peak molecular weights (*M_p_*) of the duplicate injections of system suitability solution do not differ by more than 5% of the upper value.

### 3.3. Phase 2: Comparison of Different Chromatographic Methods

In Phase 2 of the study 12 different GPC methods were compared in a single laboratory. Details of the chromatographic conditions used in each of the 12 methods are given in the text of the Supplementary Materials, and in Table S5. The heparin samples characterized in Phase 2 were the same set as for Phase 1 of the study. 

#### Analysis of the Chromatographic Data

Two methods for derivation of molecular weights from chromatograms were employed. For methods 1 (the USP monograph method), 5, 6, 7, 10, 11 and 12, in which only the refractive index detector is involved , a broad standard calibration was performed using a chromatogram of the USP Heparin Sodium Molecular Weight Calibrant RS, as described for Phase 1. Methods 2, 3, 8 and 9 used both RI and Right Angle Laser Light Scattering (RALLS) detection, for which no calibrant reference standard is needed. The relationship between refractive index increment and concentration of the analyte, known as the dn/dc parameter, changed as a function of the mobile phase used, as described in Supplementary Material section. As for the Phase 1 study, the weight-average molecular weight *M_w_*, the percentage of heparin with molecular weight in the ranges 8000 to 16,000, *M_8000–16,000_*, and 16,000 to 24,000, *M_16,000–24,000_*, and the percentage of heparin with molecular weight greater than 24,000, *M_24,000_* were evaluated.

Acceptance criteria were as for Phase 1: The chromatographic system is suitable if the chromatographic profile of samples does not overlap the mobile phase peak; secondly, the *M_w_* value determined for USP Heparin Sodium Identification RS (the system suitability sample) has to be within 500 Da of the assigned value of 16,000 Da [4].

## 4. Conclusions

A panel of 20 lots of bovine mucosal heparin had average molecular weights similar to those of porcine mucosal heparin samples, but with a wider variation from sample to sample, probably reflecting differences in manufacturing methods for heparin from a single species and tissue source. Some molecular weight values fall outside current USP acceptance criteria for heparin sodium.

Bovine lung heparin samples were lower in average molecular weight than mucosal heparin, as has been reported in the past. 

Alternative SEC methods for molecular weight analysis of heparin sodium give varying degrees of comparability with the USP monograph method. Use of the USP Heparin Sodium Molecular Weight Calibrant RS with long-lived polymer based columns gave comparable results with the USP monograph method for samples within the current acceptable range of heparin sodium samples; some methods using polymer-based columns with light scattering detection gave good agreement throughout the full range of heparin samples investigated in this study.

## Figures and Tables

**Table 1:** Phase 2: Weight-average molecular weight (*M_w_*, Da), percent proportion of material above 24,000 Da (*M_24,000_*) and the ratio *M_8000–16,000_*/*M_16,000–24,000_* (Ratio) of USP Heparin Sodium Identification RS, as measured using 12 distinct chromatographic methods. Results refer to the mean values of duplicate injections. Values were rounded to the nearest 100 Da.

Value	Chromatographic Methods (see Text and Table S5)											
	1	2	3	4	5	6	7	8	9	10	11	12
*M_w_* (kDa)	15.8	17.7	15.7	15.2	16.7	16.1	17.0	15.8	15.7	16.0	16.2	16.4
*M_24,000_*	8.8	16.86	8.93	8.17	13.39	9.12	14.79	8.96	8.47	7.93	6.20	8.32
Ratio	1.89	0.99	1.82	1.97	1.49	1.64	1.48	1.72	1.82	1.37	1.24	1.15

## Supplementary Materials

Supplementary Materials are available online. 1. Table S1: Heparin sample codes and origin; 2. Table S2A–C: Molecular weight measurements from the Phase 1 study; 3. Further details of materials and methods; 4. Table S3A–C: Molecular weight measurements for the Phase 2 study; 5. Table S4: Broad standard table for the USP Heparin Sodium Molecular Weight Calibrant RS; 6. Table S5: Summary of methods used for Phase 2.
