# Supplementary material for: Molecular Weights of Bovine and Porcine Heparin Samples: Comparison of Chromatographic Methods and Results of a Collaborative Survey
Source: Molecules. 2017 Jul 19;22(7):1214. doi: 10.3390/molecules22071214 (PMC6152300; doi:10.3390/molecules22071214)
Supplement: Supplementary file 1 [file molecules-22-01214-s001.pdf]

Molecular weights of bovine and porcine heparin samples: comparison of chromatographic methods and results of a collaborative survey.

Sabrina Bertini[1], Giulia Risi[1,2], Marco Guerrini[1] Kevin Carrick[3], Anita Y. Szajek[3,4], Barbara Mulloy[5],

1. Istituto di Ricerche Chimiche e Biochimiche "G. Ronzoni" via G. Colombo 81, 20133 Milano, Italy. 2. Department of Chemistry, University of Pavia, viale Taramelli 12, 27100 Pavia (Italy) 3. U.S. Pharmacopeial Convention (USP), 12601 Twinbrook Parkway, Rockville, MD 20852, USA. 4. Center for Scientific Review (CSR), National Institutes of Health, 6701 Rockledge Dr. Rm. 4187, Bethesda, MD 20892, USA. Institute of Pharmaceutical Sciences. 5. Franklin Wilkins Building, King's College London, 150 Stamford St. London SE1 9NH, UK.

Supplementary Material

Contents

1. Table S1: Bovine Heparin samples codes, species and tissue of origin, and donor number.

2. Phase 1: molecular weight results from a collaborative study involving six laboratories, using the chromatographic method described in the USP Heparin Sodium monograph. Table S2: A) Weight-average molecular weight M_w, b) percent proportion of high molecular weight material (M_{24000}), and c) ratio $M_{8000\text{-}16000}/M_{16000\text{-}24000}$ for 20 heparin sodium samples from bovine intestinal mucosa, two from bovine lung and two from porcine intestinal mucosa.

3. Phase 2 Further details of Materials and Methods

4. Table S3: A) Weight-average molecular weight M_w, b) percent proportion of high molecular weight material >24,000 g/mol (M_{24000}), and c) ratio ($M_{8000\text{-}16000}/M_{16000\text{-}24000}$) determined by 12 methods listed in Table S3 for 20 heparin sodium samples from bovine intestinal mucosa, two from bovine lung and two from porcine intestinal mucosa.

5. Table S4: Broad Standard Table for the USP Heparin Sodium Molecular Weight Calibrant RS

6. Table S5: Summary of methods used for Phase 2.

1. Table S1: Heparin sample codes and origin.

Heparin Sample Code	Species and tissue	Donor
Heparin Sodium Batch A-3	Porcine intestine	7
Heparin Sodium Batch D-1	Porcine intestine	7
Heparin Sodium Batch E-1	Bovine intestine	1
Heparin Sodium Batch E-2	Bovine intestine	6
Heparin Sodium Batch F-1	Bovine intestine	1
Heparin Sodium Batch F-2	Bovine intestine	6
Heparin Sodium Batch G-1	Bovine intestine	1
Heparin Sodium Batch G-2	Bovine intestine	6
Heparin Sodium Batch H-1	Bovine intestine	1
Heparin Sodium Batch H-2	Bovine lung	5
Heparin Sodium Batch K-1	Bovine intestine	3
Heparin Sodium Batch K-2	Bovine lung	5
Heparin Sodium Batch L-1	Bovine intestine	3
Heparin Sodium Batch M-1	Bovine intestine	3
Heparin Sodium Batch N-1	Bovine intestine	3
Heparin Sodium Batch O-1	Bovine intestine	3
Heparin Sodium Batch O-2	Bovine intestine	4
Heparin Sodium Batch P-1	Bovine intestine	3
Heparin Sodium Batch P-2	Bovine intestine	4
Heparin Sodium Batch Q-1	Bovine intestine	3
Heparin Sodium Batch Q-2	Bovine intestine	4
Heparin Sodium Batch R-1	Bovine intestine	3
Heparin Sodium Batch R-2	Bovine intestine	4
Heparin Sodium Batch S-1	Bovine intestine	3

2. Phase 1: molecular weight results from a collaborative study involving six laboratories, using the chromatographic method described in the USP Heparin Sodium monograph. Samples codes are as listed in Table S-1. Table S2A Weight-average molecular weight M_w(Da)

Lab	E-1	E-2	F-1	F-2	G-1	G-2	H-1	H-2	K-2	D-1
A	14700	13800	15600	14600	15800	14100	15900	12900	13400	16200
B	15100	14400	15700	15100	15700	14300	15700	13400	13700	15900
C	14900	14000	15800	14900	15500	14000	15600	14450	13300	16000
D	15000	14400	15600	15100	15800	14400	15800	13500	13800	15900
E	15300	13900	15900	14600	16100	14600	16000	13600	14000	16100
F	14800	14000	15500	14700	15600	14200	15400	13100	13600	15800
**Mean**	**14967**	**14083**	**15683**	**14833**	**15750**	**14267**	**15733**	**13492**	**13633**	**15983**
SD	197.2	233.9	134.4	213.4	189.3	197.2	197.2	490.3	235.7	134.4
RSD (%)	1.3	1.7	0.9	1.4	1.2	1.4	1.3	3.6	1.7	0.8

Lab	A-2	K-1	L-1	M-1	N-1	O-1	O-2	P-1	P-2	Q-1	Q-2	R-1	R-2	S-1
A	15600	15800	15000	15400	16300	16200	17100	20100	17900	17600	19400	17700	20800	17500
B	16100	16100	15300	15600	16800	16600	17800	20900	18475	18200	20400	16000	21600	18000
C	15700	16300	15500	15800	16900	16500	18200	20500	18700	18000	20200	18300	22100	18000
D	16000	16400	15400	15500	16800	16500	17900	20500	19000	18300	20600	18300	22400	18100
E	16000	16200	15300	15600	16900	16700	18000	20800	18800	18100	20300	18300	22000	18000
**Mean**	**15880**	**16160**	**15300**	**15580**	**16740**	**16500**	**17800**	**20560**	**18575**	**18040**	**20180**	**17720**	**21780**	**17920**
SD	193.9	205.9	167.3	132.7	224.5	167.3	374.2	280.0	377.5	241.7	411.8	890.8	552.8	213.5
RSD (%)	1.2	1.3	1.1	0.9	1.3	1.0	2.1	1.4	2.0	1.3	2.0	5.0	2.5	1.2

Table S2B Proportion of high molecular weight materialM_{24000} %

Lab	E-1	E-2	F-1	F-2	G-1	G-2	H-1	H-2	K-2	D-1
A	9	7	11	8	11	8	11	7	8	10
B	10	9	12	11	12	10	12	9	10	8
C	9	8	11	10	11	8	11	9	8	10
D	10	9	11	10	12	10	12	9	10	8
E	10	8	12	9	12	10	12	9	10	9
F	9	8	11	9	11	9	10	8	9	8
**Mean**	**9.5**	**8.2**	**11.3**	**9.5**	**11.5**	**9.2**	**11.3**	**8.5**	**9.2**	**8.8**
SD	0.50	0.69	0.47	0.96	0.50	0.90	0.75	0.76	0.90	0.90
RSD (%)	5.3	8.4	4.2	10.1	4.3	9.8	6.6	9.0	9.8	10.2

Lab	A-3	K-1	L-1	M-1	N-1	O-1	O-2	P-1	P-2	Q-1	Q-2	R-1	R-2	S-1
A	8	11	8	9	11	11	16	24	18	16	23	16	26	16
B	9	13	9	10	13	12	19	28	21	17	27	18	31	18
C	9	13	10	11	14	13	20	27	22	18	26	19	31	19
D	9	13	9	10	13	12	19	27	22	18	27	18	31	18
E	9	13	9	10	13	12	20	28	22	17	27	18	32	17
**Mean**	**8.8**	**12.7**	**9.2**	**10.2**	**12.8**	**12.1**	**18.7**	**26.7**	**21.2**	**17.2**	**26.0**	**17.6**	**30.2**	**17.5**
SD	0.34	0.84	0.68	0.71	0.87	0.65	1.37	1.44	1.45	0.94	1.70	1.02	1.95	1.05
RSD (%)	12.8	10.5	10.7	11.1	11.0	11.3	12.3	14.6	12.0	12.0	13.2	12.8	12.9	11.5

Table S2C Ratio $M_{8000\text{-}16000}/M_{16000\text{-}24000}$

Lab	E-1	E-2	F-1	F-2	G-1	G-2	H-1	H-2	K-2	D-1
A	3.1	3.6	2.9	3.2	2.7	3.7	2.7	4	3.9	1.9
B	2.1	2.5	2	2.2	1.9	2.5	1.9	2.5	2.5	1.5
C	2.1	2.4	1.8	2.2	1.9	2.5	1.9	1.5	2.5	1.4
D	2.2	2.5	2	2.3	1.9	2.5	1.9	2.6	2.5	1.5
E	2.1	2.7	1.9	2.4	1.8	2.4	1.8	2.5	2.5	1.3
F	2.2	2.5	2	2.3	1.9	2.6	1.9	2.6	2.5	1.5
**Mean**	**2.30**	**2.70**	**2.10**	**2.43**	**2.02**	**2.70**	**2.02**	**2.62**	**2.73**	**1.52**
SD	0.36	0.41	0.37	0.35	0.31	0.45	0.31	0.73	0.52	0.19
RSD (%)	15.7	15.3	17.4	14.4	15.3	16.7	15.3	27.9	19.1	12.3

Lab	A-3	K-1	L-1	M-1	N-1	O-1	O-2	P-1	P-2	Q-1	Q-2	R-1	R-2	S-1
A	1.9	2.4	2.7	2.6	2.0	2.1	2.3	1.2	2.1	1.7	1.8	1.6	1.6	1.8
B	1.4	2.0	2.2	2.1	1.6	1.6	1.7	0.8	1.6	1.3	1.3	1.2	1.2	1.4
C	1.6	1.9	2.0	1.9	1.5	1.6	1.7	1.0	1.6	1.3	1.3	1.3	1.2	1.4
D	1.4	1.9	2.1	2.0	1.5	1.6	1.7	0.9	1.6	1.2	1.3	1.2	1.2	1.3
E	1.4	1.9	2.2	2.1	1.6	1.6	1.7	0.9	1.6	1.3	1.3	1.2	1.2	1.4
**Mean**	**1.54**	**2.03**	**2.24**	**2.14**	**1.64**	**1.70**	**1.81**	**0.96**	**1.70**	**1.35**	**1.39**	**1.31**	**1.28**	**1.46**
SD	0.20	0.21	0.24	0.24	0.18	0.19	0.22	0.14	0.20	0.16	0.18	0.17	0.17	0.17
RSD (%)	12.8	10.5	10.7	11.1	11.0	11.3	12.3	14.6	12.0	12.0	13.2	12.8	12.9	11.5

3. Phase 2 Further Details of Materials and Methods.

Materials

Polyethylene oxide was obtained from Agilent Technologies; SEC columns were obtained from Tosoh Bioscience, with the exception of PLS5030 silica columns provided by Malvern Instruments. Mobile phase reagents were obtained from Sigma Aldrich.

Methods

The12 GPC methods compared are summarized in Table S5. , using Broad Standard calibration Refractive Index) or multi-detector calibration (Light Scattering). All 24 heparin samples were analysed using each of the 12 methods.

Equipment

The HPLC equipment consisted of a Viscotek system equipped with a Knauer Smartline 5100 pump, a Biotech Degasser model 2003 and a HTA autosampler model HT310L. The detector system used in this study was a Viscotek mod.305 Triple Detector Array, with a temperature controlled oven compartment set at 30 or 40 °C (see Table S5) that contains space for up to three separation columns and the detectors. Right angle laser light scattering (RALLS) is the first detector after the columns, with the following technical specifications: a 90° angle geometry for maximum signal to-noise; cell volume of 10 μL; maximum backpressure on cell of 5 psi; maximum signal of 2.5 V; a 670 nm laser light source. Refractive index (RI) is the second detector with the following technical specifications: cell volume of 12 μL; maximum backpressure on cell of 5 psi; maximum signal of 2.5 V; light emitting diode (LED) at 660 nm wavelength. Viscometer is the last detector, characterized by four capillaries (0.01"id x 24"L) with a differential Wheatstone bridge configuration.

Columns and chromatographic conditions

Both silica and polymeric columns were used, two columns in series, in particular TSKG2500PWXL + TSKG3000PWXL (polymeric), TSKG4000PWXL + TSKG3000PXL (polymeric), 2 x PLS5030(silica) and TSKG4000SWXL + TSKG3000SWXL (silica columns were preceded by a TSKGel Guard Column SWXL), at different temperatures (30 and 40°C) and injection volume (20 and 100 μl). Two mobile phase were tested: 0.1 M ammonium acetate + 0.02 % sodium azide and 0.1 M sodium nitrate + 0.05 % sodium azide.

Each sample was injected twice and the mean results of the weight-average molecular weight (M_w, Da), the percentage of heparin with molecular weight in the range 8,000 to 16,000, $M_{8000-16000}$, the percentage of heparin with molecular weight in the range 16,000 to 24,000, $M_{16000-24000}$, and the percentage of heparin with molecular weight greater than 24,000, M_{24000} were measured and are reported in Tables S3.

For methods Refractive Index method in Table S5, the broad standard USP Heparin Sodium Molecular Weight Calibrant RS was used to perform the chromatographic conventional calibration; in contrast, the Light Scattering detector does not require a chromatographic calibration, because the molecular weight distribution obtained is absolute. Themulti-detector method requires only an instrumental calibration for the determination of the detector constants; in this case, a polyethylene oxide (PEO) standard, of known M_w, polydispersity, and intrinsic viscosity was used. When the Light Scattering detector is involved, two different dn/dc

values used: a value of 0.13 for 0.1 M sodium nitrate + 0.05 % sodium azide as mobile phase, and 0.128 for 0.1 M ammonium acetate + 0.02 % sodium azide as mobile phase; both dn/dc values were experimentally determined in the Phase 2 laboratory. Chromatographic profiles were elaborated using suitable GPC software: for methods 1, 3, 4, 8 and 9,Clarity version 6.1 was used, and for methods 2, 5, 6, 7, 10, 11 and 12 OmniSEC version 4.6.2 was used.

4. Phase 2: Molecular weight results for 24 heparin samples listed in Table S1, measured using the USP Heparin Sodium monograph method (Method 1) and 11 other distinct chromatographic methods as listed in Table S5.

Table S3: A) Weight-average molecular weight M_w determined by 12 methods and 4 chromatographic column types listed in Table S3. Values differing from those determined by Method 1 by more than +/-500 are shaded

	Methods											
	1	2	3	4	5	6	7	8	9	10	11	12
Column	A	B	C	C	C	C	C	D	D	D	D	D
Heparin E-1	14656	16620	14689	14227	15457	14662	15805	14652	14628	14836	15105	15266
Heparin F-1	15631	17516	15251	14720	16117	15266	16421	15610	15116	15349	15475	15887
Heparin G-1	15828	17720	15712	15071	16272	15326	16621	15681	15474	15524	15724	16085
Heparin H-1	15911	17618	15387	14948	16186	15425	16562	15680	15358	15485	15661	15894
Heparin E-2	13845	15721	13984	13555	14678	14066	14975	13849	13786	14135	14054	14392
Heparin F-2	14606	16627	14401	14215	15568	14893	15882	14643	14620	14807	15033	15250
Heparin G-2	14120	15882	13934	13612	14696	14022	14988	14044	13850	13728	14169	14322
Heparin H-2	12870	14916	12604	12590	14254	13640	14478	12879	12931	13632	13755	14108
Heparin K-2	13377	15502	12980	12772	14568	14071	14886	13282	13056	14000	14344	14489
Heparin D-1	16168	18144	16018	15431	16550	16126	16986	16123	15811	15937	16196	16616
Heparin K-1	15838	N.A.	15587	15541	16689	15959	17083	15871	15956	16015	16076	16643
Heparin L-1	15022	N.A.	14854	14784	15781	15117	16060	15093	15136	15046	15326	15692
Heparin M-1	15425	N.A.	15168	14957	16199	15589	16555	15429	15341	15490	15706	16073
Heparin N-1	16333	N.A.	16382	16021	17279	16708	17731	16614	16424	16624	16813	17154
Heparin O-1	16245	N.A.	16161	15918	17031	16460	17454	16398	16335	16408	16528	16952
Heparin P-1	20081	N.A.	19830	19558	21077	20188	21502	20582	20090	20435	20584	21154
Heparin Q-1	17614	N.A.	17599	16982	18381	17759	18914	17675	17563	17851	17814	18352
Heparin R-1	17695	N.A.	17476	17072	18562	18278	19090	18036	17698	17915	18069	18578
Heparin S-1	17495	N.A.	17353	16987	18354	18195	18818	17807	17565	17798	17864	18260
Heparin O-2	17138	N.A.	17017	16405	18795	18525	19331	17207	17090	18062	18333	18890
Heparin P-2	17932	N.A.	17977	16892	19646	19619	20300	17987	17640	18874	19048	19650
Heparin Q-2	19414	N.A.	19442	18290	21151	21007	21944	19686	19570	20694	20478	21396
Heparin R-2	20801	N.A.	20615	19753	23354	23114	24319	21049	21126	22614	22793	23592
Heparin A-2	15579	N.A.	15546	15244	16549	16430	17060	15659	15625	16075	16168	16613

Table S3 B): percent proportion of high molecular weight material (M_{24000}) determined by 12 methods and 4 chromatographic column types listed in Table S5. Values differing from those determined by Method 1 by more than +/-10% are shaded.

	Methods											
	1	2	3	4	5	6	7	8	9	10	11	12
Column	A	B	C	C	C	C	C	D	D	D	D	D
Heparin E-1	8.51	14.61	8.62	7.80	12.57	9.54	13.37	8.78	8.80	8.94	8.44	10.11
Heparin F-1	10.73	16.93	10.18	9.05	14.19	11.03	15.32	10.69	10.15	10.64	9.68	11.79
Heparin G-1	11.17	18.80	11.21	9.59	14.20	10.85	15.42	11.10	10.70	10.70	10.08	12.28
Heparin H-1	11.36	18.37	10.48	9.49	14.27	11.03	15.53	11.12	10.60	10.92	9.83	12.03
Heparin E-2	7.09	13.91	7.72	6.89	11.37	8.49	12.04	7.81	7.60	8.23	6.96	8.75
Heparin F-2	8.44	16.28	8.64	8.19	12.96	9.91	14.08	9.37	9.18	9.56	8.95	10.43
Heparin G-2	8.04	14.08	8.09	7.39	11.70	8.74	12.52	8.51	8.17	8.11	7.95	9.29
Heparin H-2	6.75	14.68	6.21	6.26	12.36	9.73	13.16	7.27	7.23	9.14	8.91	10.53
Heparin K-2	7.87	15.70	6.88	6.98	13.52	11.36	14.54	8.23	7.84	10.83	10.92	12.10
Heparin D-1	9.80	16.91	10.08	8.49	13.71	9.21	15.38	9.38	8.26	7.36	7.04	8.91
Heparin K-1	11.03	N.A.	10.77	10.55	15.76	12.30	17.02	11.66	11.57	12.03	11.41	13.60
Heparin L-1	8.27	N.A.	8.26	8.05	12.84	9.62	13.79	8.82	8.74	8.47	8.38	10.08
Heparin M-1	9.15	N.A.	8.94	8.72	13.77	10.49	14.90	9.80	9.46	9.40	9.55	11.26
Heparin N-1	11.32	N.A.	11.80	10.89	16.30	12.06	17.68	12.31	11.72	11.54	10.72	12.72
Heparin O-1	10.97	N.A.	11.20	10.31	15.52	11.63	17.09	11.71	11.50	10.78	10.07	12.15
Heparin P-1	23.85	N.A.	23.38	22.40	29.07	24.08	30.58	26.13	24.82	25.26	23.90	27.55
Heparin Q-1	15.45	N.A.	15.63	14.10	19.33	15.67	21.45	15.98	16.16	15.40	14.22	17.09
Heparin R-1	15.69	N.A.	15.26	14.19	20.26	17.63	21.73	16.88	16.05	15.60	14.77	17.45
Heparin S-1	15.50	N.A.	15.32	14.22	20.13	17.96	21.50	16.65	16.01	16.00	15.01	17.70
Heparin O-2	16.05	N.A.	15.94	14.35	23.23	21.15	24.09	17.00	16.57	19.71	19.80	21.59
Heparin P-2	18.35	N.A.	18.95	15.88	25.28	24.55	26.97	19.36	18.49	22.51	22.29	24.63
Heparin Q-2	22.55	N.A.	23.02	19.82	29.54	28.00	31.24	24.09	23.53	27.18	26.57	29.11
Heparin R-2	26.26	N.A.	26.11	23.61	34.90	33.63	36.14	28.10	27.66	31.86	32.28	34.30
Heparin A-2	8.15	N.A.	8.89	8.22	12.85	10.66	15.34	8.64	8.24	7.93	6.68	9.48

Table S3 C): ratio ($M_{8000\text{-}16000}/M_{16000\text{-}24000}$) determined by 12 methods and 4 chromatographic column types listed in Table S5.

	Methods											
	1	2	3	4	5	6	7	8	9	10	11	12
Column	A	B	C	C	C	C	C	D	D	D	D	D
Heparin E-1	3.10	1.60	2.44	2.78	2.09	2.29	1.97	2.63	2.68	2.19	2.30	1.93
Heparin F-1	2.78	1.50	2.35	2.52	1.80	2.13	1.85	2.32	2.43	1.99	1.97	1.79
Heparin G-1	2.66	1.49	2.16	2.39	1.77	2.06	1.78	2.22	2.30	1.92	1.83	1.68
Heparin H-1	2.64	1.24	2.28	2.42	1.78	1.97	1.78	2.22	2.32	1.85	1.84	1.73
Heparin E-2	3.55	1.71	2.83	3.09	2.17	2.31	2.16	2.98	3.08	2.44	2.50	2.24
Heparin F-2	3.18	1.54	2.70	2.79	1.97	2.19	1.93	2.65	2.75	2.17	2.18	1.97
Heparin G-2	3.68	1.59	2.99	3.19	2.17	2.34	2.09	3.10	3.20	2.57	2.55	2.27
Heparin H-2	4.04	1.85	3.63	3.59	2.14	2.14	2.11	3.36	3.40	2.25	2.24	2.13
Heparin K-2	3.85	1.85	3.51	3.47	2.15	2.10	2.10	3.23	3.27	2.18	2.18	2.08
Heparin D-1	1.85	1.03	1.72	1.94	1.49	1.57	1.52	1.71	1.78	1.32	1.30	1.13
Heparin K-1	2.45	N.A.	2.31	2.35	1.88	2.03	1.85	2.28	2.33	2.01	1.96	1.76
Heparin L-1	2.69	N.A.	2.51	2.58	2.08	2.45	2.02	2.51	2.53	2.48	2.33	2.00
Heparin M-1	2.59	N.A.	2.44	2.46	1.99	2.28	1.95	2.37	2.44	2.14	2.24	1.92
Heparin N-1	1.99	N.A.	1.83	1.94	1.71	1.80	1.71	1.77	1.84	1.65	1.56	1.41
Heparin O-1	2.08	N.A.	1.94	2.03	1.70	1.84	1.78	1.85	1.93	1.67	1.65	1.40
Heparin P-1	1.21	N.A.	1.14	1.20	1.04	0.87	1.08	1.03	1.06	0.81	0.64	0.69
Heparin Q-1	1.67	N.A.	1.54	1.67	1.32	1.46	1.52	1.49	1.54	1.26	1.18	1.15
Heparin R-1	1.63	N.A.	1.57	1.65	1.42	1.30	1.49	1.46	1.50	1.23	1.13	1.08
Heparin S-1	1.78	N.A.	1.67	1.76	1.45	1.43	1.50	1.58	1.64	1.35	1.29	1.25
Heparin O-2	2.26	N.A.	2.05	2.18	1.67	1.56	1.67	2.04	2.09	1.62	1.65	1.55
Heparin P-2	2.11	N.A.	1.84	2.08	1.52	1.48	1.76	1.90	1.96	1.62	1.57	1.52
Heparin Q-2	1.76	N.A.	1.55	1.73	1.28	1.19	1.54	1.55	1.61	1.28	1.20	1.22
Heparin R-2	1.61	N.A.	1.46	1.55	1.18	1.07	1.40	1.41	1.46	1.17	1.10	1.09
Heparin A-2	1.91	N.A.	1.85	1.98	1.43	1.46	1.53	1.71	1.86	1.34	1.24	1.14

5. Table S4. Broad Standard Table for the USP Heparin Sodium Molecular Weight Calibrant RS

MW (Da)	% below MW	% above MW
6000	3.2	96.8
8000	10.4	89.6
10000	19.8	80.2
12000	31.7	68.3
14000	43.4	56.5
16000	55.5	44.5
18000	66.0	34.0
20000	74.4	25.6
22000	80.3	19.7
24000	84.4	15.6
26000	87.5	12.5
28000	90.1	9.9
32000	93.4	6.6
36000	95.6	4.4
40000	97.0	3.0

6. Table S5. Summary of methods used for phase 2. Method 1 is the reference method, taken from the USP Heparin Sodium monograph.

Method	Column Set*	Mobile Phase	Temperature	Injection Volume	Detector
1	A	0.1 M NH_4Ac + 0.02 % NaN_3	30 °C	20 μl	Refractive Index
2	B	0.1 M NH_4Ac + 0.02 % NaN_3	30 °C	100 μl	Light Scattering
3	C	0.1 M NH_4Ac + 0.02 % NaN_3	30 °C	20 μl	Light Scattering
4	C	0.1 M $NaNO_3$ + 0.05 % NaN_3	30 °C	20 μl	Light Scattering
5	C	0.1 M $NaNO_3$ + 0.05 % NaN_3	40 °C	100 μl	Refractive Index
6	C	0.1 M NH_4Ac + 0.02 % NaN_3	30 °C	100 μl	Refractive Index
7	C	0.1 M NH_4Ac + 0.02 % NaN_3	40 °C	100 μl	Refractive Index
8	D	0.1 M NH_4Ac + 0.02 % NaN_3	30 °C	20 μl	Light Scattering
9	D	0.1 M $NaNO_3$ + 0.05 % NaN_3	30 °C	20 μl	Light Scattering
10	D	0.1 M $NaNO_3$ + 0.05 % NaN_3	40 °C	100 μl	Refractive Index
11	D	0.1 M NH_4Ac + 0.02 % NaN_3	30 °C	100 μl	Refractive Index
12	D	0.1 M NH_4Ac + 0.02 % NaN_3	40 °C	100 μl	Refractive Index

* TSKG4000SWXL + TSKG3000SWXL = A; 2 x PLS5030 = B; TSKG2500PWXL + TSKG3000PWXL = C; TSKG4000PWXL + TSKG3000PWXL = D
